# Neighborhood privilege and environmental conditions in urban parks: an analysis across the 24 most populated urban areas in the United States

**DOI:** 10.1088/1748-9326/ae5e93

**Published:** 2026-04-28

**Authors:** Greta K Martin, Tsz-Kin Siu, William P Klein, Rachel Clark, Kelvin C Fong

**Affiliations:** 1 Department of Environmental and Occupational Health, The George Washington University Milken Institute School of Public Health, 950 New Hampshire Ave NW, Washington, DC, 20037, United States of America; 2 Department of Earth and Environmental Sciences, Dalhousie University, 6287 Alumni Cres, Halifax, Nova Scotia B3H 4R2, Canada; 3 Trust for Public Land, 100 M St SE #900, Washington, DC 20003, United States of America; 4 R Clark Consulting, Washington, DC 20002, United States of America

**Keywords:** urban parks, air pollution, fine particulate matter, nitrogen dioxide, normalized difference vegetation index, wet-bulb globe temperature, index of concentration at the extremes

## Abstract

Urban parks provide mental and physical health benefits by mitigating harmful environmental exposures, including heat and air pollution. Urban parks often cluster in more privileged neighborhoods. Health burdens from harmful environmental exposures are highest in less privileged ones. Using Trust for Public Land’s *ParkServe* database of publicly accessible parks, sociodemographic data from the American Community Survey, and high-resolution satellite-imagery, we compared park size and environmental conditions by neighborhood privilege. To ascertain privilege, we calculated the combined race and income index of concentration at the extremes (ICEs) in each census tract of the 24 most populous urban areas in the United States (U.S.). We found that parks in the least privileged neighborhoods (i.e. bottom quartile of ICE) had on average 1.24 ppb higher concentrations of nitrogen dioxide (NO_2_) and 1.00 *μ*g/m^−3^ higher fine particulate matter (PM_2.5_) than parks in the most privileged neighborhoods (top ICE quartile). Parks in the most privileged neighborhoods were significantly larger and cooler in more than half of the included urban areas and significantly greener in over a third. Our results reveal disparities in the environmental conditions, and thus expected health benefits, between parks in the most and least privileged neighborhoods in the most populous urban areas in the U.S.

## 1. Introduction

Urban greenspaces (e.g. parks) are associated with improved mental and physical health through three key pathways: reducing environmental harm (i.e. less heat, noise, and air pollution), restoring capacities (i.e. improved restoration and reduced stress), and building capacities (i.e. increased physical activity and social gathering) (Markevych et al 2017). Systematic reviews have linked higher vegetation, often referred to as greenness and measured with satellite imagery, with reduced all-cause mortality (Rojas-Rueda et al 2019), cardiovascular and ischemic heart disease mortality (Bianconi et al 2023), risk of depression (Liu et al 2023), and lung and prostate cancer risk (Li et al 2023). Greenspaces can also reduce the urban heat island effect by replacing heat-absorbent surfaces like asphalt and concrete with dirt and vegetation. A multi-country study found that a 20% increase in greenspace was associated with a 9.02% (95% confidence interval [CI]: 8.88, 9.16) decrease in urban heat-related mortality (Choi et al 2022). A health impact assessment across 93 European cities found that increasing tree-canopy coverage to 30% could reduce summer deaths among adults by 2644 (95% CI: 2444, 2824) (Iungman et al 2023). Greenspaces may also reduce exposures to harmful air pollutants, including fine particulate matter (PM_2.5_) and nitrogen dioxide (NO_2_) due to lower traffic in these areas and through the direct removal by vegetation (Markevych et al 2017). A recent study in Brazil found that PM_2.5_ reductions accounted for roughly 50% of the estimated effect of greenspace on hospitalizations for respiratory conditions and more than 75% of those for circulatory diseases (Requia et al 2025). Another study from China found that reductions in PM_2.5_ and NO_2_ accounted for 23.80% and 26.60% of the impact of greenness on mortality among diabetics, respectively (Wu et al 2024). There is some evidence that the size, shape, and distribution of greenspaces can impact their effect on public health. A systematic review of greenspace morphology found that larger, more connected, and more irregularly shaped greenspaces had stronger positive associations with health outcomes (Wang et al 2024). In particular, the size of the largest greenspace in a given area was associated with decreased mortality (Wang et al 2024). Urban greenspaces, particularly larger and more vegetated ones, can reduce harmful environmental exposures and improve public health.

While greenspaces identified using satellite imagery include all vegetation, including privately owned spaces such as golf courses and residential gardens, publicly accessible urban parks provide a more accurate measure of population exposure to urban greenspaces. Higher quality parks (i.e. greener, cooler, less polluted, and larger) are likely to convey greater health benefits. Therefore, urban park creation could be particularly well-suited for addressing environmental disparities in the United States (U.S.), as historically underserved communities are less green, and have higher burdens of air pollution and heat. One study comparing the average 2010 greenness of urban neighborhoods by Home Owners’ Loan Corporation grades, a discriminatory designation of the 1930s informing mortgage lending, found that neighborhoods that received an ‘A’ grade were nearly 25% greener than those with a ‘D’ grade (Nardone et al 2021). Similar racial and ethnic disparities exist in urban air pollution and heat exposures. Compared to the 2019 national average, NO_2_ was 10%–15% higher in census tracts with higher Black, Hispanic, Asian and multiracial populations, compared to 5%–15% lower in tracts with majority non-Hispanic white populations (Kerr et al 2023). Similarly, zip-codes with higher proportions of Black or low-income residents experienced significantly higher 2010 average PM_2.5_ levels (Knobel et al 2023). A study of urban heat stress disparities found that a $10 000 increase in census-tract-level median per capita income was associated with a median heat index decrease of 0.56 °C (Chakraborty et al 2023). The authors also observed a 0.04 °C median heat index increase for every percentage higher of a census tract’s Black population (Chakraborty et al 2023). Stark intra-city environmental exposure disparities exist in the U.S. by race/ethnicity and income.

Expanding parks in underprivileged neighborhoods could provide important health and equity benefits. However, not all parks provide equivalent health benefits. In this study, we explore whether parks located in more privileged census tracts differ from those in less privileged tracts by factors that mediate the relationship between greenspace and health including environmental conditions (greenness, air quality, and heat) and size (percentage park and size of the largest intersecting park within each census tract). We quantify differences in park size and environmental quality by measures of neighborhood privilege to examine whether the public health benefits of urban U.S. parks differ by race and income.

## 2. Methods

We analyzed park characteristics by census tract across the 24 most populous urban areas of the U.S. We considered park environmental conditions: ambient air quality (nitrogen dioxide [NO_2_] and fine particulate matter [PM_2.5_]), greenness (normalized difference vegetation index [NDVI]), and heat (wetbulb globe temperature [WBGT]), and size: the percentage of census tract area that is a park and the area of its largest intersecting park. We compared mean park characteristics across census tract quartiles of privilege.

### 2.1. Neighborhood privilege: Index of Concentration at the Extremes (ICE)

ICE is a measure of concentrated privilege and disadvantage (Massey 2001). It is associated with health outcomes, including pre-term birth and infant mortality (Chambers et al 2019), fatal and non-fatal assaults (Krieger et al 2017), hypertension (Feldman et al 2015), breast cancer-specific survival (Goel et al 2022), and HIV outcomes (Gant Sumner et al 2025). Previous studies have used ICE measures of income, race and ethnicity, as well as combined measures of income and race (Feldman et al 2015, Krieger et al 2016, 2017, Chambers et al 2019, Goel et al 2022, Gant Sumner et al 2025) and found that ICE measures perform best at the census tract level (Krieger et al 2017). ICE ranges from −1 (entirely least advantaged group) to 1 (entirely most advantaged group). A census tract with a value of 0 could indicate no residents from either extreme, or equal numbers from both extremes (figure S1).

We retrieved 5 year estimates from the 2019 to 2023 American Community Survey (ACS) to calculate ICE metrics (U.S. Census Bureau 2023). We used ‘B03002: Hispanic or Latino Origin by Race’ to calculate ICE race/ethnicity, ‘B19001: Household income in the past 12 months (in 2024 inflation-adjusted dollars)’ to calculate ICE income, and tables ‘B19001B: Household income in the past 12 months (in 2024 inflation-adjusted dollars) (Black or African American Alone Householder)’ and ‘B19001H: Household income in the past 12 months (in 2024 inflation-adjusted dollars) (While Alone, Not Hispanic or Latino Householder)’ to calculate a combined measure of ICE race and income (equation (1)). The combined measure of ICE race and income for each census tract (*i*) is calculated as follows:

(1) $$\text{Index of Concentration at the Extremes (ICE)}\;\;=\frac{{\text{most privileged}}_{i}-{\text{least privileged}}_{i}}{{\text{total population}}_{i}}.$$

We defined the most privileged group as the total number of non-Hispanic white householders earning $200 000 or more in the past 12 months (Table B19001H) and the least privileged group as the sum of Black householders in each income bucket from less than $10 000 up to $35 000–$39 999 in the past 12 months (Table B19001B). We used the income table for all races combined (Table B19001) to identify the total population. While there are other racial identities with substantial urban populations, we have limited our analysis to these groups because they had sufficient population sizes across *all* 24 urban areas. Furthermore, it was not possible to compare high-income non-Hispanic White householders to low-income Black *or* Latino householders, as the B19001 tables are not split out by combined race and ethnicity.

Prior studies using ICE income measures have defined the extremes as the 20th and 80th percentiles of income (Feldman et al 2015, Krieger et al 2016, 2017, Chambers et al 2019). To determine these income percentiles, we subset the 2023 ACS to all counties within our 24 urban areas of interest (appendix, table S1). Using the entire 24-urban area population for comparability, we then determined population counts in each of the income brackets defined by ACS table B19001. Next, we calculated a running sum by income bracket, to determine which to include in the top and bottom ~20% of the population. On the low end, including households with incomes less than $40 000 accounted for 21% of the total urban population. On the high end, including households with incomes of at least $200 000 accounted for 18.7% of the total urban population. In a sensitivity analysis, we use city-specific cut points to determine the 20th and 80th percentiles of income, to account for income differences across urban areas in the U.S. (appendix, figures S2-S4 and table S2).

### 2.2. Park & urban area definitions

We used the Trust for Public Land’s ParkServe database to identify parks and their spatial boundaries (Trust for Public Land 2024). ParkServe is a comprehensive database comprising over 150 000 parks, inclusive of outdoor named destinations that encourage public activities such as socializing, enjoying nature, or play/exercise, across all urban areas in the U.S. We identified the 25 most populous U.S. cities (U.S. Census Bureau 2024), which equated to 24 urban areas, as defined by the U.S. Census Bureau (appendix, figure S5) (Dallas and Fort Worth, TX both independently rank in the top 25 most populous cities but are considered under the same urban area). We used the 2020 Census relationship file to identify counties included in each urban area definition (U.S. Census Bureau 2020). We then selected the census tracts within these counties using the *tidycensus* package in R. Next, we performed a spatial join between tract and park geometries to separate out the park sections falling within each census tract, accounting for parks that crossed administrative boundaries. For all environmental exposures (NO_2_, PM_2.5_, NDVI, and WBGT), we calculated the average area-weighted values of all park sections within each census tract.

### 2.3. Greenness: Normalized Difference Vegetation Index (NDVI)

We measured greenness using NDVI, a satellite-derived measure ranging from −1 to 1, with negative values indicating water and ice, values near zero representing built-up areas, and higher positive values denoting greener, denser vegetation (‘NASA Earth Observatory,’ 2000). We used the European Space Agency (ESA)’s Sentinel-2 satellite imagery, accessed through Google Earth Engine (European Space Agency 2025). These data have a 10 m x 10 m resolution and capture the same geographic area every five days. We used all images with <20% cloud coverage captured between April 1st and October 31st of years 2019–2023, corresponding to the growing season of the ACS data collection period. We used images from all years, rather than just 2021, because NDVI can fluctuate substantially from year to year, driven primarily by precipitation (Martin et al 2025). We masked cloudy pixels in each image. We then used the near infrared and red (Red) bands to calculate NDVI as follows:

NDVI=(NIR−Red)∕(NIR+Red).

After averaging NDVI across all 2019–2023 growing season images, we removed pixels covered by water for more than nine months of the year using ESA’s 2020 landcover dataset (European Space Agency 2020).

### 2.4. Heat: Wet-Bulb Globe Temperature (WBGT)

We used WBGT, a ‘real feel’ measure of heat comprising temperature, humidity, wind speed, and solar radiation (Ahn et al 2024). We combined temperature and humidity variables from the Parameter-Elevation Relationships on Independent Slopes Model (PRISM) dataset at its highest resolution (800 m × 800 m) (Oregon State University 2025) with solar radiation and wind speed variables from ERA5-Land (European Centre for Medium-Range Weather Forecasts Climate Reanalysis) (Muñoz Sabater 2019), resampled from the native resolution of 0.1° × 0.1° to the PRISM grids.

Following previously established methods (Liljegren et al 2008, Tuholske et al 2021), we calculated the 2021 April 1st to October 31st WBGT average, using the R package ‘HeatStress’ (Casanueva et al 2019, Ahn et al 2024).

### 2.5. Air pollution: fine particulate matter (PM_2.5_) & nitrogen dioxide (NO_2_)

We considered two air pollutants, PM_2.5_ and NO_2_. Ambient PM_2.5_ was the fourth leading risk factor for premature deaths worldwide according to the 2025 State of Global Air report (Health Effects Institute 2025). PM_2.5_ is associated with respiratory and cardiovascular diseases, type 2 diabetes, adverse birth outcomes, and cancers and is responsible for the largest burden of air pollution-related mortality and morbidity (Health Effects Institute 2025). NO_2_ is a traffic-related pollutant linked to increased asthma incidence and exacerbations (Health Effects Institute 2025).

We used PM_2.5_ data (*μ*g/m^−3^) from the Washington University of St. Louis (Shen et al 2024). These data combine NASA satellite products, the GEOS-CHEM chemical transport model, and ground monitors, and are provided at the 0.01° × 0.01° resolution (approximately one square kilometer). We selected the 2021 annual average, representing the midpoint of our neighborhood privilege measure.

We retrieved NO_2_ data from Tropospheric Monitoring Instrument (TROPOMI) from the NASA Health Air Quality Applied Science Team at 0.01° × 0.01°, using ESA’s version 2.4 (Goldberg 2024). We converted tropospheric vertical column densities (1 × 10^15^ molecules/cm^2^) to surface concentrations (ppb) following established methods (Cooper et al 2020), which differentiate clean and polluted areas using vertical NO_2_ and meteorology profiles. We accessed vertical profile data from the GEOS Composition Forecasts, a system assimilating GEOS-Chem model outputs at 0.25° × 0.25° with 72 layers (Knowland et al 2022). We calculated annual average surface NO_2_ concentrations for 2021.

### 2.6. Size: percentage park & area of largest intersecting park

To calculate the percentage park of each census tract, we divided each ParkServe park into sections, based on census tract boundaries. We then calculated the area of each section. Next, we summed the areas of all park sections within each census tract and divided this sum by the total tract area.

To determine the size of the largest park that touches a given census tract, we used the st_intersects() function from the ‘*sf*’ R package to obtain a list of all parks that intersect with each census tract. We then selected the park with the greatest area.

Finally, we averaged the tract percentage park and the area of its largest intersecting park by ICE quartile to compare park size metrics by neighborhood privilege.

### 2.7. Statistical analysis

We ranked each urban area’s census tracts into quartiles of privilege (combined income and race ICE). We then calculated the mean environmental and size characteristics of parks for each quartile across the 24 urban areas. Using *t*-tests, we compared the mean park values within census tracts in an urban area’s lowest and highest ICE quartiles. We used a *p*-value of <0.05 when evaluating statistical significance.

## 3. Results

**Figure 1.**
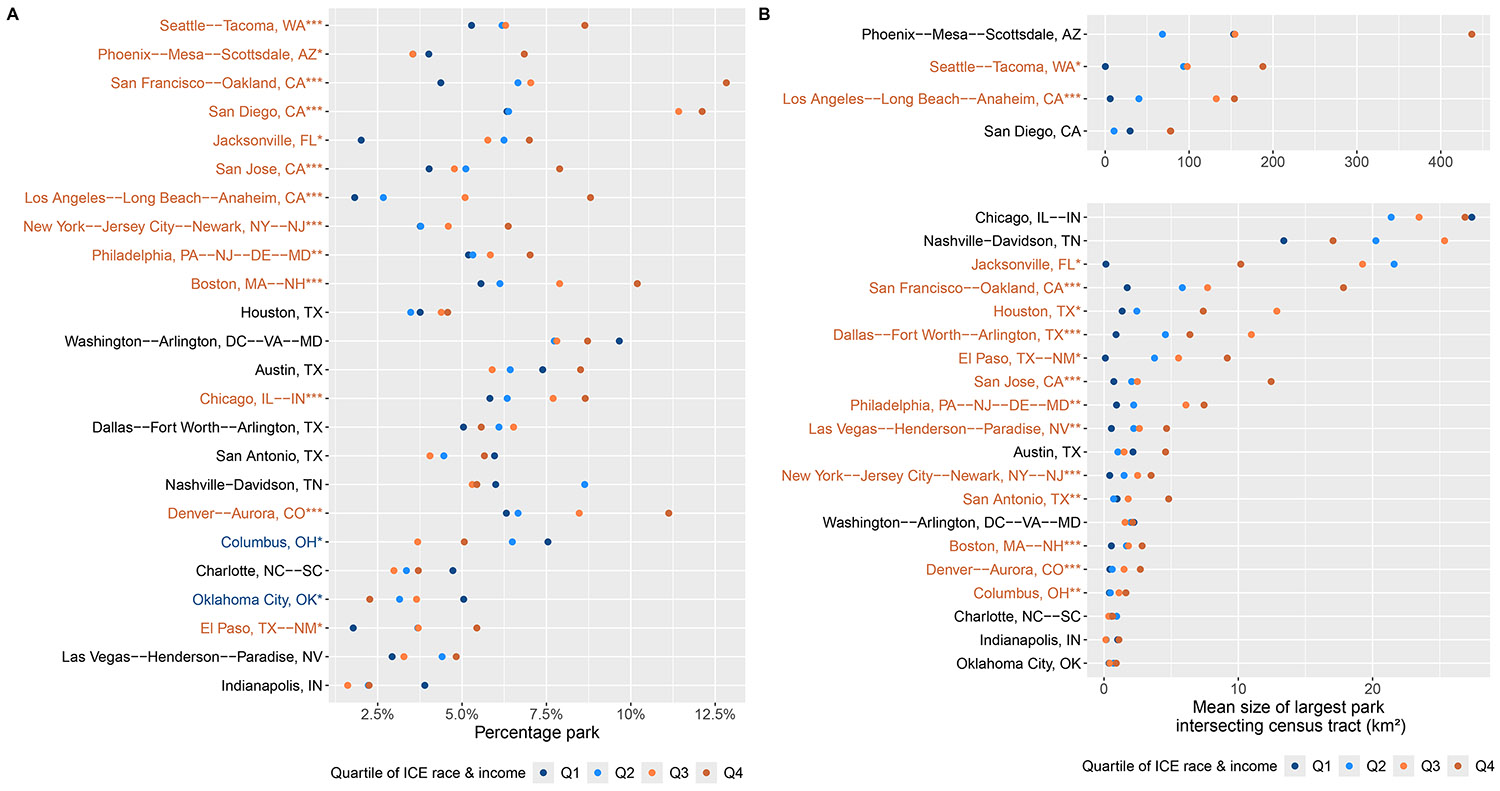
Percentage park (Panel A) and average size of the largest park intersecting any census tract (Panel B) by quartile of race and income index of concentration at the extremes (ICE). Each dot is colored by ICE race and income quartile and represents the average value within census tracts in that quartile. Urban areas are sorted from greatest to smallest overall percentage park (Panel A) or largest intersecting park (Panel B) and their names are displayed in orange if the *t*-test comparing the most privileged quartile (*Q*4) was significantly higher than that of the least privileged quartile (*Q*1) and displayed in blue if the opposite was true. The number of stars represents the level at which these differences are statistically significant (***p < .001, **p < .01, *p < .05). Four urban areas are displayed with a separate *x*-axis scale to account for the comparatively large size of their parks.

We found large differences within and across urban areas in percentage park (figure 1(A)). Publicly accessible parks covered on average 8.7% of the 24 most populous urban areas, ranging from <1% in Indianapolis, IN to 37.5% in Seattle-Tacoma, WA (appendix, figure S6). Across these urban areas, the least privileged neighborhoods by race and income had 2.1 percentage points less park area as a percent of land area. Roughly half of the included locations had significantly higher percentages of parks in the most versus least privileged quartiles of census tracts, while the opposite was true in Columbus, OH and Oklahoma City, OK. California had the largest disparities across ICE quartiles. The difference in percentage park between the most and least privileged neighborhoods was 3.9%, 5.8%, 7.0%, and 8.5% in San Jose, San Diego, Los Angeles–Long Beach–Anaheim, and San Francisco–Oakland, respectively.

The median size of the largest park intersecting any census tract across the urban areas was 4.3 km^2^, ranging from 0.59 km^2^ in Oklahoma City, OK to 200.05 km^2^ in Phoenix–Mesa–Scottsdale, AZ (appendix, figure S7). Compared with the percentage park metric, there were more urban areas with significant disparities in park size (figure 1(B)). In 15 of the 24 urban areas, residents of the most privileged neighborhoods had access to a significantly larger park than those in the least privileged. On average, the largest park in the top quartile of ICE was 3.97 km^2^ larger than in the bottom quartile (range: −0.50 km^2^ to 284 km^2^ across urban areas). Differences in percentage park and the size of the largest intersecting park were similar by quartiles of ICE race/ethnicity and ICE income (appendix, figures S4 and S5).

**Figure 2.**
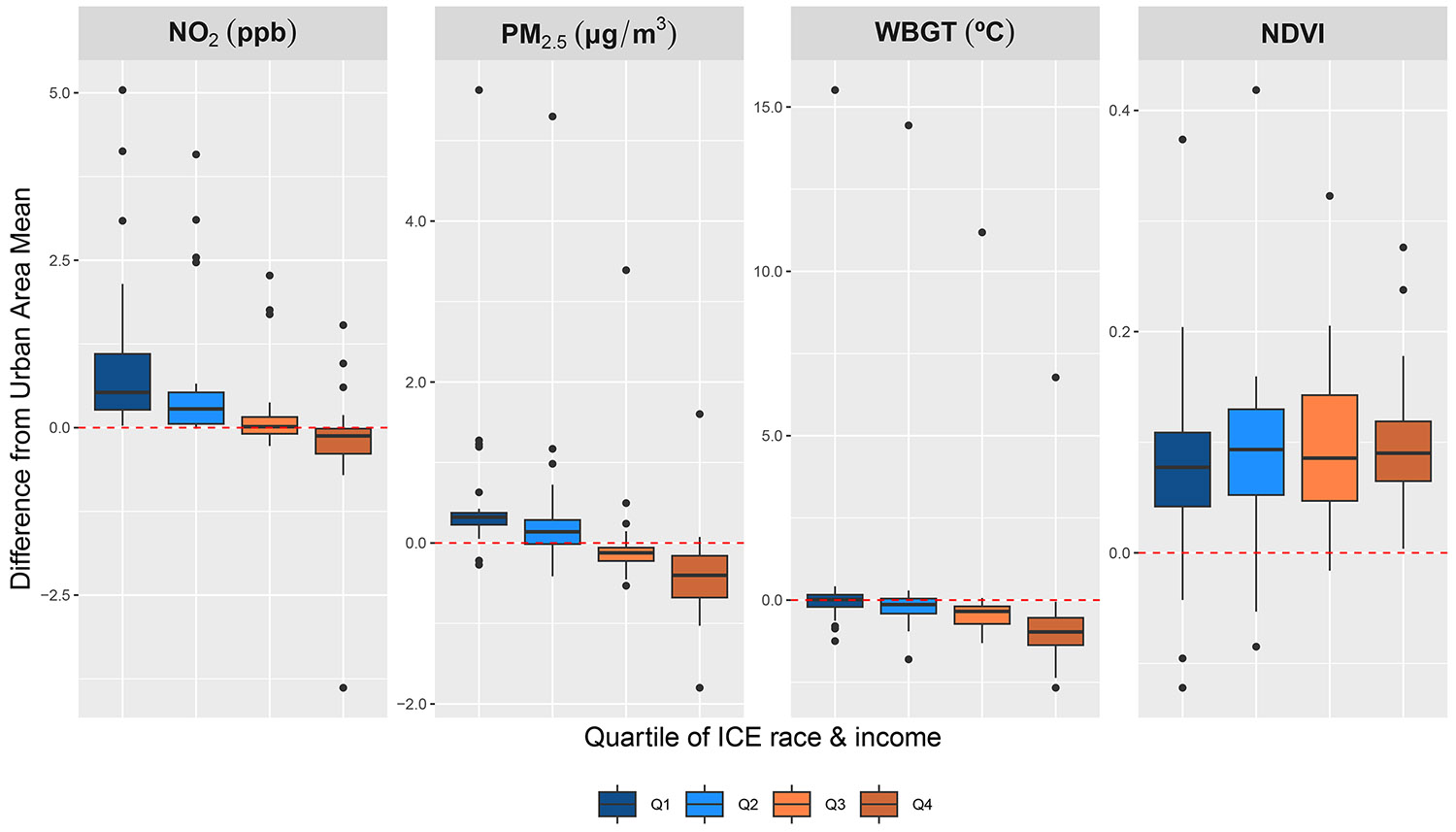
Park environmental characteristics by quartile of race and income index of concentration at the extremes (ICE). Each boxplot is colored by ICE quartile, from least (*Q*1) to most (*Q*4) privileged, and shows the difference in the average environmental exposure level of parks in that quartile from the urban area mean. Each box spans the 25th to 75th percentile of data, with the median value marked by the solid line. The whiskers of the box plot extend to the minimum and maximum values, except for outliers, which are shown with dots. Zero, or equivalency between the urban area mean and the mean value of parks in that ICE quartile, is marked by a red dotted line.

Neighborhood privilege was strongly associated with park air pollution, and to a lesser extent heat (figure 2). The median NO_2_ and PM_2.5_ levels of parks located in the bottom two quartiles of neighborhood privilege exceeded their urban area average, while median air pollution levels in the top two quartiles were lower or the same as their citywide average. The median NO_2_ difference between parks and the urban average was 0.53, 0.28, −0.01, and −0.12 ppb, from least to most privileged quartile. In New York–Jersey City–Newark, NY-NJ; San Francisco–Oakland, CA; Chicago, IL-IN; and Los Angeles–Long Beach–Anaheim, CA, parks in the bottom two quartiles of privilege had over 2 ppb higher NO_2_ than the urban average. In contrast, parks in the most privileged census tracts of Los Angeles–Long Beach–Anaheim, CA had almost 4 ppb lower NO_2_ than the urban mean. The median PM_2.5_ difference between parks and the urban area mean was 0.32, 0.14, −0.12, and −0.40 *μ*g/m^−3^, from least to most privileged neighborhood quartile. The most privileged neighborhoods of Los Angeles–Long Beach–Anaheim, CA remained a negative outlier, with 1.8 *μ*g/m^−3^ lower PM_2.5_ than the urban area average. Park PM_2.5_ in San Francisco–Oakland, CA exceeded the urban average across all levels of privilege; the average park concentrations in least to most privileged neighborhoods were 5.6, 5.3, 3.4, and 1.6 *μ*g/m^−3^ higher than the urban mean.

Generally, parks across all levels of neighborhood privilege were greener and cooler than their urban average (figure 2). The median NDVI difference was very similar across privilege quartiles (0.08–0.09). Parks in the most privileged neighborhoods had less spread in NDVI difference from the urban mean (range: 0.00–0.28), indicating the health and density of vegetation in these parks were more similar than those in the less privileged areas (range: −0.12 to 0.37). The median WBGT of parks consistently decreased from least to most privileged neighborhoods; the median difference from the urban area mean by ICE quartile was 0.01, −0.14, −0.34 and −0.97 °C, respectively. Results by ICE race/ethnicity and ICE income followed similar patterns (appendix, figures S6 and S7).

**Figure 3.**
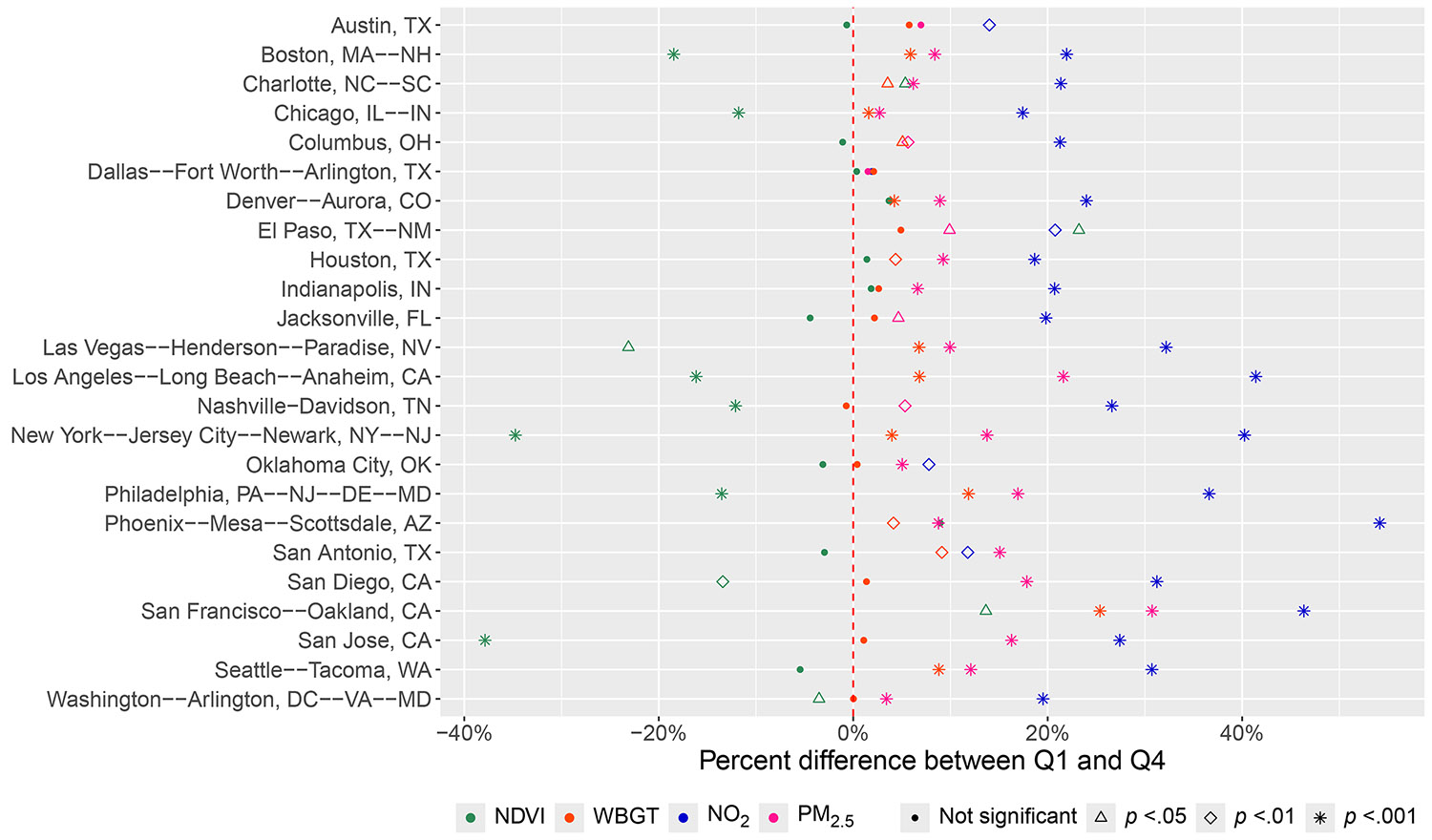
Percent difference in environmental conditions between the least (*Q*1) and most (*Q*4) privileged neighborhoods (ICE race and income) by urban area. Each color represents an environmental exposure. The shape reflects the significance of the *t*-test between *Q*1 and *Q*4. Equivalency between the most and least privileged neighborhoods is shown by the red dotted line. Symbols to the right (left) of this line indicate that *Q*1 values were higher (lower) than *Q*4 values.

In general, we found that parks were less polluted and cooler in the most versus least privileged neighborhoods (figure 3). This pattern was most consistent in air pollution, where NO_2_ and PM_2.5_ concentrations were significantly lower in the top ICE quartile compared to the bottom quartile in 23 and 22 of the 24 most populous urban areas, respectively. Park NO_2_ was over 30% higher in the least versus most privileged neighborhoods of Phoenix–Mesa–Scottsdale, AZ; San Francisco–Oakland, CA; Los Angeles–Long Beach–Anaheim, CA; New York–Jersey City–Newark, NY-NJ; Philadelphia, PA-NJ-DE-MD; Las Vegas–Henderson–Paradise, NV; San Diego, CA; and Seattle–Tacoma, WA. Park PM_2.5_ was over 30% higher in San Francisco–Oakland, CA. The mean absolute difference in air pollution between the bottom and top ICE quartiles was 1.24 ppb (range: 0.04, 6.03) for NO_2_ and 1.00 *μ*g/m^−3^ (0.13–4.03) for PM_2.5_ (appendix, figure S7). There was not a significant difference in park NO_2_ or PM_2.5_ by neighborhood privilege in Dallas–Fort Worth–Arlington, TX.

The association between neighborhood privilege and park greenness was less clear. Park NDVI was significantly higher in neighborhoods in the top quartile of privilege compared to the bottom quartile in 10 of the 24 included urban areas, and significantly lower in Charlotte, NC-SC; El Paso, TX-NM; and San Franciso–Oakland, CA. Parks in the top quartile of neighborhood privilege were over 30% greener than those in the least privileged quartile in New York–Jersey City–Newark, NY-NJ and San Jose, CA. The mean absolute difference in park NDVI between the top and bottom ICE quartiles was 0.03 (range: −0.10, 0.15) (appendix, figure S7). Considering temperature, parks in the most privileged neighborhoods were significantly cooler than those in the least privileged in 14 of the 24 included urban areas. The mean park temperature was over 20% higher in the least versus most privileged census tracts in only one urban area (San Francisco–Oakland, CA). The mean absolute difference in WBGT was 1.3 °C between the bottom and top ICE quartiles (range: −0.17, 8.74). Parks were 1 ° C hotter in the least privileged neighborhoods compared to the most privileged ones in half of the urban areas. While the magnitudes of disparities in park environmental conditions varied by individual city, the overall trends were similar using ICE income and ICE race/ethnicity (appendix, figures 8 and 9).

## 4. Discussion

More privileged neighborhoods generally had access to larger parks, and these parks had more favorable environmental conditions. We observed the most consistent trends when looking at air pollution, where the parks in the least privileged quartile had, on average, 1.24 ppb higher NO_2_ and 1.00 *μ*g/m^−3^ higher PM_2.5_ concentrations. We found mixed results when comparing the percentage park, greenness, and heat. Parks in the most privileged neighborhoods were significantly cooler in over half of the 24 urban areas and significantly greener in more than a third. Half of the urban areas had significantly higher park percentages in their most privileged census tracts, while the percentage park was significantly higher in the least privileged tracts of two urban areas. Our findings suggest that there are disparities between the most and least privileged neighborhoods in the environmental quality, and thus health benefits, of parks.

Discriminatory U.S. practices may explain why parks in less privileged neighborhoods are generally smaller, hotter, more polluted, and less green. Redlining, the practice of denying people mortgages based on their race and ethnicity, began in the 1930s and remained common policy until it was deemed illegal by the Fair Housing Act of 1968. In addition to its impacts on home ownership and the accumulation of wealth, redlining has left lasting effects on the built environment. The Mapping Inequality project notes that neighborhoods with poor Homeowner’s Loan Corporation grades (C and D) were described at the time of assessment as having more paved surfaces and being in closer proximity to manufacturing, while neighborhoods receiving A and B grades were referred to with words like ‘wooded’, ‘shade’, and ‘trees’ (Hoffman 2024). These existing disparities in proximity to sources of pollution and parks were exacerbated by ‘urban renewal’ and highway programs that explicitly targeted ‘slums’ and African American communities to clear land for the construction of highways, hospitals, universities, and offices (Rothstein 2017). While parks might serve as a haven from the pollution, heat, and concrete of the urban landscape, our findings show that disparities in environmental conditions, previously documented at the neighborhood level, persist in urban parks. In a sensitivity analysis, we compared the mean environmental conditions of land not covered by a park to the park mean by ICE quartile (appendix, figure S15). We found this difference tended to be larger in the bottom ICE quartile for air pollution and temperature, and smaller for NDVI. In other words, parks were more effectively mitigating heat and air pollution in less privileged neighborhoods, though these differences were small across all quartiles. On the other hand, parks were greener relative to their surroundings in more privileged neighborhoods.

Urban parks could help reduce environmental and health disparities. Past studies have shown that residential proximity to parks is more strongly associated with positive health outcomes in lower socioeconomic areas and locations with worse air pollution and less greenspace. A systematic review concluded that access to greenspace had a stronger association with positive health outcomes among individuals of lower socioeconomic status (Rigolon et al 2021). While greenspace may mitigate the impacts of harmful environmental factors like heat and air pollution, studies have also identified greenspace as an effect modifier of health damages from these factors. Studies of urban areas in England (Alcock et al 2017) and Philadelphia, Pennsylvania (Yen et al 2025) have found that tree density was more strongly associated with lower rates of asthma exacerbations in areas with high levels of PM_2.5_ and NO_2_. In Seoul, Korea, mortality risk for a 1 °C increase in heat above the 90th percentile (25.1 °C) was nearly twice as strong in neighborhoods in the bottom third of NDVI compared to those in the top third (Son et al 2016). Urban vegetation can provide important health benefits, with evidence suggesting that benefits are strongest where existing greenspace is limited and air pollution is high.

Our study has its limitations motivating future work. First, while we use urban area definitions from the U.S. Census Bureau’s urban area to county crosswalk, some of these counties also capture rural or suburban areas. This may skew our results by including parks that are less accessible to the urban core. Second, despite using the highest resolution environmental data available, our exposure estimates in small park sections may include adjacent built-up land, leading to measurement error. Third, NDVI has its limitations. It does not classify greenness into vegetation types (i.e. forest, grass), which could be helpful for urban planning and design. Additionally, in warmer, more humid climates, increased vegetation may increase humidity along with pest populations. Fourth, we used one year of data from the midpoint of the census data to capture WBGT. If 2021 consisted of temperature anomalies, our estimates might not be reflective of urban heat. While this choice may impact comparisons across locations, it is unlikely to impact the intra-city analysis of ICE quartiles. Fifth, environmental harm is only one of the theorized healthpromoting pathways of parks (Markevych et al 2017). Building capacities, such as space for gathering and exercising, and restoring capacities, such as reducing stress, also link parks to improved health. While we examine greenness using NDVI, which is a known indicator of mental stress relief, there are likely other elements of the park environment which strengthen or diminish its restoration capabilities. For example, noise, vegetation type, and the presence of water may also influence stress. Similarly, we do not capture any park elements related to social gathering or physical activity. There may be tradeoffs in these pathways. Parks with more open fields may allow for more physical activity but provide less shade and be less green. Greenways alongside major roads may encourage more active transportation but expose commuters to higher levels of air pollution. The addition of infrastructure like paved pathways, playgrounds, and basketball courts may facilitate social gathering and physical activity but exacerbate heat exposure by creating more impervious surfaces. By measuring only one of the hypothesized causal pathways between greenspaces and health, our results do not capture the full picture of the disparities that exist in the health promotion of parks by neighborhood privilege. Finally, we examine how size and environmental conditions of parks differ across neighborhoods, but the presence of a nearby park does not guarantee its access or use. Some parks within a census tract may be hard to get to, while others outside the tract may be more easily accessible, for example due to a highway impeding pedestrian access or walking paths facilitating it. The findings of our study reflect the unique social and historical realities of the U.S. and are not necessarily generalizable to cities outside of the U.S.

While this study does not address all park characteristics that influence health, it highlights inequities in park environmental and size characteristics, particularly in air pollution and park area. Expanding parks may improve mental and physical health, especially in lower socioeconomic areas. However, simply adding parks in disadvantaged neighborhoods will not eliminate environmental disparities. To maximize park health benefits, other aspects of the built environment must be considered. Solutions like the ‘expressway teardown movement’ provide a possible blueprint (Mohl 2012). Several U.S. cities including Boston, MA; New York City, NY; and Portland, OR have removed inner-city highways, placing roadways further from urban residents. These projects, which moved roads underground or further from the urban core, can reduce traffic-related air pollution and provide opportunities to create parks or greener residential communities using the land previously occupied by highways. While such interventions are costly and time-consuming, smaller-scale changes, like planting trees and adding infrastructure to encourage social gathering and physical activity, can help improve park quality. In future work, we plan to examine how these park characteristics relate to one another, to better understand how environmental conditions mediate the relationship between parks and health and be able to recommend which park characteristics are most health beneficial.

## 5. Conclusion

In the 24 most populous urban areas, more privileged neighborhoods had access to larger parks with cleaner air. Parks in the most privileged neighborhoods had lower concentrations of air pollution by on average 1.24 ppb NO_2_ and 1.00 *μ*g/m^−3^ PM_2.5_ compared those in the least privileged areas. In over half of the 24 most populous urban areas, these parks were also significantly cooler and made up a greater percentage of the neighborhood. While parks can help to mitigate urban environmental harms, efforts should be made to ensure that more, and better quality, parks are created in less privileged neighborhoods to reduce existing environmental disparities.

## Supplementary Material

Supplementary Material

Supplemental material available at: https://doi.org/10.1088/1748-9326/ae5e93/data1.

Supplementary material for this article is available online

## Data Availability

The data that support the findings of this study are openly available at the following URLs: ParkServe: https://www.tpl.org/park-data-downloads, ACS: https://data.census.gov/, NDVI: https://developers.google.com/earth-engine/datasets/catalog/COPERNICUS_S2_SR_HARMONIZED WBGT: https://prism.oregonstate.edu/, PM_2.5_: https://sites.wustl.edu/acag/surface-pm2-5/, NO_2_: https://www.earthdata.nasa.gov/data/instruments/tropomi/data-access-tools.
