# Supplementary material for: Neighborhood privilege and environmental conditions in urban parks: an analysis across the 24 most populated urban areas in the United States
Source: Environ Res Lett. Author manuscript; Available in PMC 2026 May 20. (PMC13186425; doi:10.1088/1748-9326/ae5e93)
Supplement: Supplementary Material [file NIHMS2170422-supplement-Supplementary_Material.docx]

*Neighborhood privilege and environmental conditions in urban parks: an analysis across the 24 most populated urban areas in the United States*

Greta K. Martin^1^, Tsz-kin Siu^2^, William P. Klein^3^, Rachel Clark^4^, Kelvin C. Fong^1^

^1^ Department of Environmental Health, The George Washington University Milken Institute of Public Health, 950 New Hampshire Ave NW, Washington, DC 20015, USA

^2^ Department of Earth and Environmental Sciences, Dalhousie University, 6287, Alumni Cres, Halifax, Nova Scotia, B3H 4R2, Canada

^3^ Trust for Public Land, 100 M St SE #900, Washington, DC 20003, USA

^4^ R Clark Consulting, Washington, DC, 20002, USA

**SUPPLEMENTAL MATERIAL**

**Contents of this file**

Table S1-S2

Figures S1-S15

Figure S1 provides a graphical representation of our privilege measure, the Index of Concentration at the Extremes (ICE). Table S1 contains the county names and codes used to define the 24 most populous urban area bounds and Figures S5 maps their locations. Figures S2-4 mirror Figures 1-3 of the main text for our sensitivity analysis using individual city Census data to define the 20^th^ and 80^th^ percentiles of income. The income cut points and corresponding population proportions for each city’s 20^th^ and 80^th^ income percentiles are listed in Table S2. Figures S6-7 show average size metrics for the entire urban area. Figures S8-9, S10-11, and S13-14 mirror Figures 1-3 of the main text, respectively, using ICE race/ethnicity and ICE income to define neighborhood privilege in place of the combined race and income measure used in the main text. Figure S12 shows park environmental characteristics by quartile of ICE race and income, broken out for each city individually. Finally, Figure S15 shows the difference between the non-park and park area means of each environmental exposure across levels of ICE race and income.

***Figure S1.*** The Index of Concentration at the Extremes. Dark blue figures represent persons belonging to the least privileged social group while dark orange figures represent persons belonging to the most privileged group. For example, a census tract with a value of -1 would consist entirely of Black households earning less than $40,000 a year, while a tract with a value of +1 would consist only of non-Hispanic White households making at least $200,000 a year. In the example population shown for an ICE value of 0, there is an equal number of people in the least and most privileged groups, as well as others outside these extremes.

***Table S1.*** *Urban areas included in study and their corresponding county codes.*

| Urban Area Name | Urban Area Code | County Names | County Codes |
| --- | --- | --- | --- |
| Austin, TX | 04384 | Hays County, Travis County, Williamson County | 48209, 48453, 48491 |
| Boston, MA--NH | 09271 | Bristol County, Essex County, Middlesex County, Norfolk County, Plymouth County, Suffolk County, Worcester County, Rockingham County | 25005, 25009, 25017, 25021, 25023, 25025, 25027, 33015 |
| Charlotte, NC--SC | 15670 | Gaston County, Iredell County, Mecklenburg County, Union County, York County | 37071, 37097, 37119, 37179, 45091 |
| Chicago, IL--IN | 16264 | Cook County, DeKalb County, DuPage County, Grundy County, Kane County, Kendall County, Lake County, McHenry County, Will County, Lake County, Porter County | 17031, 17037, 17043, 17063, 17089, 17093, 17097, 17111, 17197, 18089, 18127 |
| Columbus, OH | 19234 | Delaware County, Fairfield County, Franklin County, Licking County, Pickaway County, Union County | 39041, 39045, 39049, 39089, 39129, 39159 |
| Dallas--Fort Worth--Arlington, TX | 22042 | Collin County, Dallas County, Denton County, Ellis County, Johnson County, Kaufman County, Parker County, Rockwall County, Tarrant County, Wise County | 48085, 48113, 48121, 48139, 48251, 48257, 48367, 48397, 48439, 48497 |
| Denver--Aurora, CO | 23527 | Adams County, Arapahoe County, Boulder County, Broomfield County, Denver County, Douglas County, Jefferson County, Weld County | 08001, 08005, 08013, 08014, 08031, 08035, 08059, 08123 |
| El Paso, TX--NM | 27253 | Doña Ana County, El Paso County | 35013, 48141 |
| Houston, TX | 40429 | Brazoria County, Chambers County, Fort Bend County, Galveston County, Harris County, Liberty County, Montgomery County, Waller County | 48039, 48071, 48157, 48167, 48201, 48291, 48339, 48473 |
| Indianapolis, IN | 41212 | Boone County, Hamilton County, Hancock County, Hendricks County, Johnson County, Madison County, Marion County, Morgan County | 18011, 18057, 18059, 18063, 18081, 18095, 18097, 18109 |
| Jacksonville, FL | 42346 | Clay County, Duval County, St. Johns County | 12019, 12031, 12109 |
| Las Vegas--Henderson--Paradise, NV | 47995 | Clark County | 32003 |
| Los Angeles--Long Beach--Anaheim, CA | 51445 | Los Angeles County, Orange County, San Bernardino County, Ventura County | 06037, 06059, 06071, 06111 |
| Nashville-Davidson, TN | 61273 | Davidson County, Robertson County, Rutherford County, Sumner County, Williamson County, Wilson County | 47037, 47147, 47149, 47165, 47187, 47189 |
| New York--Jersey City--Newark, NY--NJ | 63217 | Bergen County, Essex County, Hudson County, Hunterdon County, Mercer County, Middlesex County, Monmouth County, Morris County, Ocean County, Passaic County, Somerset County, Sussex County, Union County, Warren County, Bronx County, Kings County, Nassau County, New York County, Putnam County, Queens County, Richmond County, Rockland County, Suffolk County, Westchester County | 34003, 34013, 34017, 34019, 34021, 34023, 34025, 34027, 34029, 34031, 34035, 34037, 34039, 34041, 36005, 36047, 36059, 36061, 36079, 36081, 36085, 36087, 36103, 36119 |
| Oklahoma City, OK | 65080 | Canadian County, Cleveland County, Logan County, Oklahoma County | 40017, 40027, 40083, 40109 |
| Philadelphia, PA--NJ--DE--MD | 69076 | New Castle County, Cecil County, Burlington County, Camden County, Cumberland County, Gloucester County, Salem County, Berks County, Bucks County, Chester County, Delaware County, Montgomery County, Philadelphia County | 10003, 24015, 34005, 34007, 34011, 34015, 34033, 42011, 42017, 42029, 42045, 42091, 42101 |
| Phoenix--Mesa--Scottsdale, AZ | 69184 | Maricopa County, Pinal County | 04013, 04021 |
| San Antonio, TX | 78580 | Bexar County, Comal County, Guadalupe County, Kendall County, Medina County | 48029, 48091, 48187, 48259, 48325 |
| San Diego, CA | 78661 | San Diego County | 06073 |
| San Francisco--Oakland, CA | 78904 | Alameda County, Contra Costa County, Marin County, San Francisco County, San Mateo County | 06001, 06013, 06041, 06075, 06081 |
| San Jose, CA | 79039 | Alameda County, San Mateo County, Santa Clara County | 06001, 06081, 06085 |
| Seattle--Tacoma, WA | 80389 | King County, Kitsap County, Pierce County, Snohomish County | 53033, 53035, 53053, 53061 |
| Washington--Arlington, DC--VA--MD | 92242 | District of Columbia, Anne Arundel County, Montgomery County, Prince George's County, Arlington County, Fairfax County, Loudoun County, Prince William County, Stafford County, Alexandria city, Fairfax city, Falls Church city, Manassas city, Manassas Park city | 11001, 24003, 24031, 24033, 51013, 51059, 51107, 51153, 51179, 51510, 51600, 51610, 51683, 51685 |

Figures S2-4 mirror the results presented in Figures 1-3 of the main text but are shown using city-specific income cut-offs to determine the ICE race and income classifications. The results from this sensitivity analysis resemble our main findings, though some statistical significance levels change across city - park characteristic pairs. In general, the differences between the top and bottom ICE quartiles were more extreme when using city-specific definitions of the 20^th^ and 80^th^ income percentiles. The income cut points and corresponding population proportions for each city’s 20^th^ and 80^th^ income percentiles are listed in Table S2.

***Figure S2.*** *Percentage park (Panel A) and average size of the largest park intersecting any census tract (Panel B) by quartile of race and income Index of Concentration at the Extremes (ICE). Each dot is colored by ICE race quartile and represents the average value within census tracts in that quartile. Urban areas are sorted from greatest to smallest overall percentage park (Panel A) or largest intersecting park (Panel B) and their names are displayed in orange if the t-test comparing the most privileged quartile (Q4) was significantly higher than that of the least privileged quartile (Q1) and displayed in blue if the opposite was true. The number of asterisks represents the level at which these differences are statistically significant (***p<.001, **p<.01, *p<.05).*

***Figure S3.*** *Park environmental characteristics by quartile of race and income Index of Concentration at the Extremes (ICE). Each boxplot is colored by ICE quartile and shows the difference in the average environmental exposure level of parks in that quartile from the urban area mean. Zero, or equivalency between the urban area mean and the mean value of parks in that ICE quartile, is marked by a red dotted line.*

**

***Figure S4.*** *Percent difference in environmental conditions between the least (Q1) and most (Q4) privileged neighborhoods (ICE race and income) by urban area.* *Each symbol represents an environmental exposure. The shape reflects the significance of the t-test between Q1 and Q4. Equivalency between the most and least privileged neighborhoods is shown by the red dotted line.*

***Table S2.*** *Income thresholds and corresponding population proportions for each urban area.*

| **Urban Area** | **20th percentile** | **80th percentile** | **Population proportion (<20^th^)** | **Population proportion (>80^th^)** |
| --- | --- | --- | --- | --- |
| Austin, TX Urban Area | <$45,000 | ≧$200,000 | 0.203 | 0.183 |
| Boston, MA--NH Urban Area | <$40,000 | ≧$200,000 | 0.198 | 0.227 |
| Charlotte, NC--SC Urban Area | <$35,000 | ≧$150,000 | 0.184 | 0.233 |
| Chicago, IL--IN Urban Area | <$35,000 | ≧$200,000 | 0.195 | 0.156 |
| Columbus, OH Urban Area | <$35,000 | ≧$150,000 | 0.196 | 0.221 |
| Dallas--Fort Worth--Arlington, TX Urban Area | <$40,000 | ≧$200,000 | 0.203 | 0.148 |
| Denver--Aurora, CO Urban Area | <$45,000 | ≧$200,000 | 0.199 | 0.185 |
| El Paso, TX--NM Urban Area | <$25,000 | ≧$125,000 | 0.223 | 0.167 |
| Houston, TX Urban Area | <$35,000 | ≧$150,000 | 0.205 | 0.237 |
| Indianapolis, IN Urban Area | <$35,000 | ≧$150,000 | 0.202 | 0.207 |
| Jacksonville, FL Urban Area | <$35,000 | ≧$150,000 | 0.211 | 0.195 |
| Las Vegas--Henderson--Paradise, NV Urban Area | <$30,000 | ≧$150,000 | 0.185 | 0.182 |
| Los Angeles--Long Beach--Anaheim, CA Urban Area | <$35,000 | ≧$200,000 | 0.190 | 0.176 |
| Nashville-Davidson, TN Urban Area | <$40,000 | ≧$150,000 | 0.203 | 0.230 |
| New York--Jersey City--Newark, NY--NJ Urban Area | <$35,000 | ≧$200,000 | 0.200 | 0.212 |
| Oklahoma City, OK Urban Area | <$30,000 | ≧$150,000 | 0.198 | 0.168 |
| Philadelphia, PA--NJ--DE--MD Urban Area | <$35,000 | ≧$200,000 | 0.204 | 0.157 |
| Phoenix--Mesa--Scottsdale, AZ Urban Area | <$40,000 | ≧$150,000 | 0.207 | 0.225 |
| San Antonio, TX Urban Area | <$30,000 | ≧$150,000 | 0.184 | 0.189 |
| San Diego, CA Urban Area | <$45,000 | ≧$200,000 | 0.212 | 0.196 |
| San Francisco--Oakland, CA Urban Area | <$50,000 | ≧$200,000 | 0.199 | 0.330 |
| San Jose, CA Urban Area | <$60,000 | ≧$200,000 | 0.212 | 0.362 |
| Seattle--Tacoma, WA Urban Area | <$50,000 | ≧$200,000 | 0.207 | 0.229 |
| Washington--Arlington, DC--VA--MD Urban Area | <$60,000 | ≧$200,000 | 0.219 | 0.279 |

***Figure S5.*** *Location of the 24 most populous urban areas in the United States.*

**

***Figure S6.*** *Percentage park of the entire urban area. Each dot represents an urban area and is colored by the overall percentage park.*

*****Figure S7.*** *Average size of the largest park intersecting any census tract within the urban area. Each dot represents an urban area and is colored by the mean area (km^2^) of all census tracts’ largest intersecting park.*

**

***Figure S8.*** *Percentage park (Panel A) and average size of the largest park intersecting any census tract (Panel B) by quartile of race Index of Concentration at the Extremes (ICE). Each dot is colored by ICE race quartile and represents the average value within census tracts in that quartile. Urban areas are sorted from greatest to smallest overall percentage park (Panel A) or largest intersecting park (Panel B) and their names are displayed in orange if the t-test comparing the most privileged quartile (Q4) was significantly higher than that of the least privileged quartile (Q1) and displayed in blue if the opposite was true. The number of asterisks represents the level at which these differences are statistically significant (***p<.001, **p<.01, *p<.05).*

**

***Figure S9.*** *Percentage park (Panel A) and average size of the largest park intersecting any census tract (Panel B) by quartile of income Index of Concentration at the Extremes (ICE). Each dot is colored by ICE income quartile and represents the average value within census tracts in that quartile. Urban areas are sorted from greatest to smallest overall percentage park (Panel A) or largest intersecting park (Panel B) and their names are displayed in orange if the t-test comparing the most privileged quartile (Q4) was significantly higher than that of the least privileged quartile (Q1) and displayed in blue if the opposite was true. The number of asterisks represents the level at which these differences are statistically significant (***p<.001, **p<.01, *p<.05).*

**

***Figure S10.*** *Park environmental characteristics by quartile of race/ethnicity Index of Concentration at the Extremes (ICE). Each boxplot is colored by ICE quartile and shows the difference in the average environmental exposure level of parks in that quartile from the urban area mean. Zero, or equivalency between the urban area mean and the mean value of parks in that ICE quartile, is marked by a red dotted line.*

**

***Figure S11.*** *Park environmental characteristics by quartile of income Index of Concentration at the Extremes (ICE). Each boxplot is colored by ICE quartile and shows the difference in the average environmental exposure level of parks in that quartile from the urban area mean. Zero, or equivalency between the urban area mean and the mean value of parks in that ICE quartile, is marked by a red dotted line.*

**

***Figure S12.*** *Park environmental characteristics across individual cities by quartile of race and income Index of Concentration at the Extremes (ICE). Each dot is colored by ICE quartile and shows the average environmental exposure level of parks in that quartile. Urban area names are displayed in orange if the t-test comparing the mean environmental exposure of parks in the most privileged quartile (Q4) was statistically higher than that of the least privileged quartile (Q1) and displayed in blue if the opposite is true. The number of asterisks represents the level at which these differences are statistically significant (***p<.001, **p<.01, *p<.05).*

**

***Figure S13.*** *Percent difference in environmental conditions between the least (Q1) and most (Q4) privileged neighborhoods (ICE race) by urban area.* *Each symbol represents an environmental exposure. The shape reflects the significance of the t-test between Q1 and Q4. Equivalency between the most and least privileged neighborhoods is shown by the red dotted line.*

***Figure S14.*** *Percent difference in environmental conditions between the least (Q1) and most (Q4) privileged neighborhoods (ICE income) by urban area.* *Each symbol represents an environmental exposure. The shape reflects the significance of the t-test between Q1 and Q4. Equivalency between the most and least privileged neighborhoods is shown by the red dotted line.*

***Figure S15.*** *Park and non-park environmental characteristics by quartile of race and income Index of Concentration at the Extremes (ICE). Each boxplot is colored by ICE quartile and shows the difference in the average environmental exposure level of parks in that quartile from the average environmental exposure level of the rest of the “non-park” census tract. Zero, or equivalency between the park and non-park area mean in that ICE quartile, is marked by a red dotted line.*
